# Graphene/ chitosan tubes inoculated with dental pulp stem cells promotes repair of facial nerve injury

**DOI:** 10.3389/fchem.2024.1417763

**Published:** 2024-06-03

**Authors:** Jingxuan Sun, Lina He, Qi An, Xu Ye, Jinjie Ma, Jing Yan, Xiaoqi Xie, Xiangyu Sun, Yumei Niu, Wenxin Cao

**Affiliations:** ^1^ The First Affiliated Hospital of Harbin Medical University, School of Stomatology, Harbin Medical University, Harbin, China; ^2^ National Key Laboratory of Science and Technology on Advanced Composites in Special Environments, Harbin Institute of Technology, Harbin, China; ^3^ Zhengzhou Research Institute, Harbin Institute of Technology, Zhengzhou, China

**Keywords:** facial nerve injury, graphene/chitosan tubes, dental pulp stem cells, facial nerve regeneration, facial nerve composite tubes

## Abstract

**Introduction:** Facial nerve injury significantly impacts both the physical and psychological] wellbeing of patients. Despite advancements, there are still limitations associated with autografts transplantation. Consequently, there is an urgent need for effective artificial grafts to address these limitations and repair injuries. Recent years have witnessed the recognition of the beneficial effects of chitosan (CS) and graphene in the realm of nerve repair. Dental pulp stem cells (DPSCs) hold great promise due to their high proliferative and multi-directional differentiation capabilities.

**Methods:** In this study, Graphene/CS (G/CST) composite tubes were synthesized and their physical, chemical and biological properties were evaluated, then DPSCs were employed as seed cells and G/CST as a scaffold to investigate their combined effect on promoting facial nerve injury repair.

**Results and Disscussion:** The experimental results indicate that G/CST possesses favorable physical and chemical properties, along with good cyto-compatibility. making it suitable for repairing facial nerve transection injuries. Furthermore, the synergistic application of G/CST and DPSCs significantly enhanced the repair process for a 10 mm facial nerve defect in rabbits, highlighting the efficacy of graphene as a reinforcement material and DPSCs as a functional material in facial nerve injury repair. This approach offers an effective treatment strategy and introduces a novel concept for clinically managing facial nerve injuries.

## Introduction

Due to factors such as trauma, tumors and iatrogenic causes, facial nerve injury is a common clinical condition in oral and maxillofacial surgery. Complete rupture of nerve fibers resulting from facial nerve defects has a significant impact on the physical and mental wellbeing of patients (Takezawa et al., 2018). Effectively repairing facial nerve injuries has always presented a major challenge for clinicians. While autologous nerve transplantation is the most effective treatment for long-distance facial nerve defects, it still faces numerous limitations, including limited donor sources and denervation in the donor area. Allogeneic nerve transplantation encounters issues with immune rejection. Although autologous blood vessels, muscles, nerve sheaths, ligaments, and other materials have shown some efficacy in nerve injury repair, they are hindered by limited availability, restricting their widespread clinical application (Modrak et al., 2020). Therefore, in recent years, finding a less invasive facial nerve repair method with comparable effectiveness to autologous nerve transplantation has emerged as a research focus (Hou et al., 2023).

In recent years, the development of optimal nerve conduit materials to facilitate facial nerve repair has emerged as a research focal point. Nerve conduits serve to bridge the severed ends of a nerve, offering adequate space for nerve regeneration. They play a crucial role in supporting nerve regeneration and promoting axon growth, while also preventing interference from surrounding tissues in the nerve regeneration process. Nerve conduits exhibit significant potential for application in facial nerve regeneration, despite the proliferation of numerous conduit designs, an ideal solution remains elusive. Hence, continued research in this domain is imperative.

CS is a kind of natural macromolecule polysaccharide with good biocompatibility, degradability and excellent biological properties. The amino group in its molecular structure makes it more reactive and easy to be chemically modified. In recent years, it has been gradually applied in the field of tissue engineering, and studies have shown that it is beneficial to the growth of nerve cells and the repair of nerve injury (Cong et al., 2024). CS tubes (CST) serve as nerve conduits have consequently demonstrated promising outcomes in nerve repair, and the potential application of CS in tissue engineering has gained recognition (Zhang et al., 2023). Our previous studies showed that CST combined with directed fibrin promoted the repair of facial nerve injury in rabbits (Mu et al., 2021). However, the efficacy of this repair was still inferior to that achieved with autologous nerve transplantation. Additionally, we observed excessive degradation of CST before completion of the repair process, the rate of degradation did not fully match the rate of nerve regeneration leading to deterioration in its mechanical properties. Consequently, it is imperative to further optimize CST to better align with the structural and functional requirements necessary for promoting nerve regeneration by combining with some reinforcing materials to enhance its mechanical strength (Jafarisavari et al., 2024).

Since the appearance of single-layer graphene in 2004 (Novoselov et al., 2004), graphene and its derived carbon materials have attracted wide attention due to their excellent physicochemical and optoelectronic properties. As a two-dimensional carbon nanomaterial, graphene has strong mechanical properties, which can provide good support strength whether used alone or in combination with other materials. In addition, graphene has excellent electrical conductivity, and its prepared materials have bioelectrical activity that is conducive to signal transmission. Therefore, it is regarded as an ideal scaffold material in the field of neural tissue engineering (Zhao et al., 2024a). Increasingly, studies have confirmed the significant potential of graphene nanotechnology in repairing peripheral nerves (Grijalvo and Díaz, 2021). Lu et al. (Zhao et al., 2024b) prepared good biocompatible graphene-based fibers with good electrical and mechanical properties, which were used to repair sciatic nerve injury in rats combined with electrical stimulation, and achieved similar therapeutic effects as autologous nerve transplantation, proving that graphene-based materials are promising methods for the treatment of peripheral nerve defects. Zhao et al. (Zhao et al., 2023) prepared chitosan/graphene oxide (GO) membranes by electrodeposition that promote the upregulation of nerve markers in Schwann cells, which they prepared as nerve guide conduit to repair a 10-mm sciatic nerve defect in rats. The results showed that the sciatic nerve function and nerve tissue recovery were improved, which was similar to the results of the autologous nerve transplantation group. It is demonstrated that graphene and its derived materials have great application potential in the field of peripheral nerve injury. However, the application of graphene in the repair of facial nerve injury is still relatively rare, and who combined with CS to repair facial nerve injury has not been reported, so the study of the effect of G/CST in the repair of facial nerve injury is necessary. In this study, graphene as reinforcement material mixed with CS to prepare composite tubes is expected to improve the physical and chemical properties of CST, thereby promoting the repair of nerve injury.

Stem cell technology can achieve the purpose of repairing the injury by transplanting stem cells to the injured site, by regulating the biological behavior of stem cells, reconstructing the structure and function of the injured site, which has great application potential in nervous system diseases. Because of its homology with Schwann cells, an important glial cell in the peripheral nervous system, DPSCs have great potential in the repair of facial nerve repair. DPSCs derived from the neural crest, and have strong proliferation and differentiation ability, which is a kind of pluripotent mesenchymal stem cells with great application value. Compared to other stem cells, DPSCs possess distinct advantages in neural differentiation (Ullah et al., 2016; Zheng et al., 2021), they can secrete neurotrophic factors (Sultan et al., 2020) to exert neuroprotective functions, which Saez et al. found to promote regeneration of the facial nerve trunk (Saez et al., 2019).

The reconstruction and recovery of tissue and function after facial nerve injury is still a major challenge. The limitation of existing treatment options leads to the urgent need to develop nerve tubes that can effectively guide facial nerve regeneration. Our previous study showed that CSTs have good biocompatibility, but the simple CSTs degrade rapidly and the mechanical properties need to be enhanced. In this study, graphene was used as a reinforcing material to prepare a composite tube with CS, the prepared G/CSTs were examined through material testing and the biocompatibility was evaluated *in vitro.* Then DPSCs were used as bioactive functional components in combination with the G/CSTs to repair facial nerve injury *in vivo*, and the reparative effects of which were investigated in order to provide novel strategies for facial nerve injury repair and instill hope in patients afflicted with such injuries. To date, there have been no reports on the assessment of utilizing G/CSTs alone or in combination with DPSCs for repairing rabbit facial nerve injuries and enhancing functional recovery.

## Materials and methods

### Preparation of the synthetic tubes

Sodium hydroxide powder (Boster) was dissolved in distilled water to obtain a solution of 1 mol/L. CS powder (Aladdin) was added to 1% aqueous acetic acid (Tianjin Tianli Chemical Reagent Co.)solution, heated and stirred in a water bath at 50°C to obtain a CS solution with a concentration of 3% (w/v). The lumbar puncture needle (Xiyanghong medical equipment, Guangzhou) was immersed in 3% CS solution to form a coating, dried naturally and then inserted into 1 mol/L sodium hydroxide solution. After repeating the above process several times, the needle was oven dried overnight at 65°C. The prepared chitosan conduits were isolated the next day by soaking in distilled water for 30 min, then dried and cutted to form a tube with an inner diameter of 1.8 mm, a length of 14 mm, and a thickness of about 1 mm, which was sterilized for use.

The reduced GO (See Supplementary Material 1.1 for the synthesis method) was prepared into a suspension of 1 mg/mL. After ultrasonic dispersion, the suspension was diluted 100 times, and then dropped onto the special copper mesh and mica sheet for transmission electron microscopy (TEM) and atomic force microscopy (AFM) to evaluate the morphology and properties of which. Then it was mixed with 3% CS solution and stirred evenly to obtain a mixed solution with a concentration of 0.075 mg/mL of graphene. Synthetic tubes for nerve regeneration were formed as described in the previous paragraph and sterilized for later use.

### Microscopic observations and structural analysis of the synthetic tubes

The above tubes were freeze-dried and were sputter-coated with a layer of Pt for 120 s. Then the microscopic morphology of the samples was observed by scanning electron microscope(SEM). The potassium bromide pellet method (Cai et al., 2022) was used to prepare the samples for Fourier transform infrared spectroscopy (FTIR) analysis. In general, the dried samples were ground and mixed with potassium bromide powder, and then pressed into thin slices in a tablet press. FTIR (NICOLET8700) was used to analyze the samples, and the infrared test range was 4,000–400 cm^−1^. The scan rate was 1 cm^−1^. Raman spectroscopy was performed using a Raman spectrometer (LabRAM HR Evolution) at a laser wavelength of 532 nm, that is, the change in the crystal structure of the tubes after mixing graphene was studied by the detection of the characteristic peak of carbon atoms. The test range is 400–3,000 cm^−1^.

### Physical and chemical properties of the synthetic tubes

The tensile strength of the conduits was tested using a universal testing machine at a tensile rate of 1 mm/min. Each group of experiments was repeated for 5 times, and the stress-strain curve was drawn to compare the mechanical properties of CST and G/CST. Subsequently, the water absorption rate of the composite tubes for 24 h and the degradation rate *in vitro* for 28 days were measured, and the specific experimental procedures are described in the Supplementary Material 1.2 and 1.3.

### Assessment of biocompatibility of the synthetic tubes *in vitro*

The related experiments in this study were approved by the Animal Ethics Committee of the First Affiliated Hospital of Harbin Medical University, and the procedures met the relevant standards.

Rabbit DPSCs (rDPSCs) were isolated and cultured by tissue block method (The specific experimental procedures are described in the Supplementary Material 1.4), SEM was used to observe the cell adhesion on the CS and G/CS film, the 3-(4,5-dimethylthiazol-2-yl)-2,5 diphenyl tetrazolium bromide (MTT) assay was used to evaluate the proliferation of cells cultured on the CS and G/CS films to detect the cytocompatibility of G/CS film, and Calcein-AM/PI Double Stain Kit (Yeasen) was used for cell viability assay (Supplementary Material 1.5).

### Application of CST and G/CST *in vivo*

Animal surgical procedures were carried out according to Guides for the Care and Use of Laboratory Animals from the Chinese Ministry of Public Health and U.S. National Institutes of Health. 25 healthy New Zealand white rabbits weighted 2.3–3.5 kg were provided by the First Affiliated Hospital of Harbin Medical University. The rabbits had normal habits and no abnormal movements of facial muscles and whiskers, then devided into five groups: empty CS tube group (CST group), empty graphene/CS tube group (G/CST group), CS tube loaded with DPSCs group (CST + DPSCs group), graphene/CS tube loaded with DPSCs group (G/CST + DPSCs group) and the autograft group (Autograft group). After anesthesia, the right cheek branch of the facial nerve was isolated, and 7 mm of the nerve was cut off, and the nerve stumps were retracted to form a 10 mm facial nerve defect model. Five groups of nerve conduits were transplanted into the defect, and the recovery of facial nerve function and histological regeneration were observed at corresponding time points. (The specific experimental procedures are described in the Supplementary Material.)

### Statistical analysis

In this study, all data were reported as mean standard deviation and evaluated using one-way or two-way analysis of variance (ANOVA). Each experiment was carried out at least thrice.

## Results and discussion

### Morphological characteristics of graphene and composite tubes

Facial nerve injury significantly affects both the physical and mental health of patients (Radwan et al., 2019). Autologous nerve transplantation is commonly utilized in clinics to repair injuries; however, it is beset by significant limitations (Markiewicz et al., 2021). Therefore, it is necessary to find an effective treatment method comparable to autologous nerve repair (Pan et al., 2020). CS is a natural polysaccharide with good biocompatibility and has been shown to promote the adhesion and growth of nerve cells (Şahin et al., 2022; Xue et al., 2023), which emerges as an outstanding scaffold material for facial nerve tissue engineering. However, we have observed limitations with the efficacy of simple CST, as their rapid degradation impedes their ability to maintain requisite structural integrity throughout the nerve repair process. This is also a criticism of many natural polymer materials, and often can be mixed with other materials to prepare composite materials to improve their physical and chemical properties. Studies have demonstrated that nerve scaffolds fabricated from CS-based composite materials exhibit remarkable performance and favorable mechanical properties, rendering them suitable as scaffolds for nerve tissue engineering (Wu et al., 2015). Therefore, our efforts have focused on identifying mechanical reinforcement materials to enhance simple CST and meet the demands of facial nerve repair.

**FIGURE 1:**
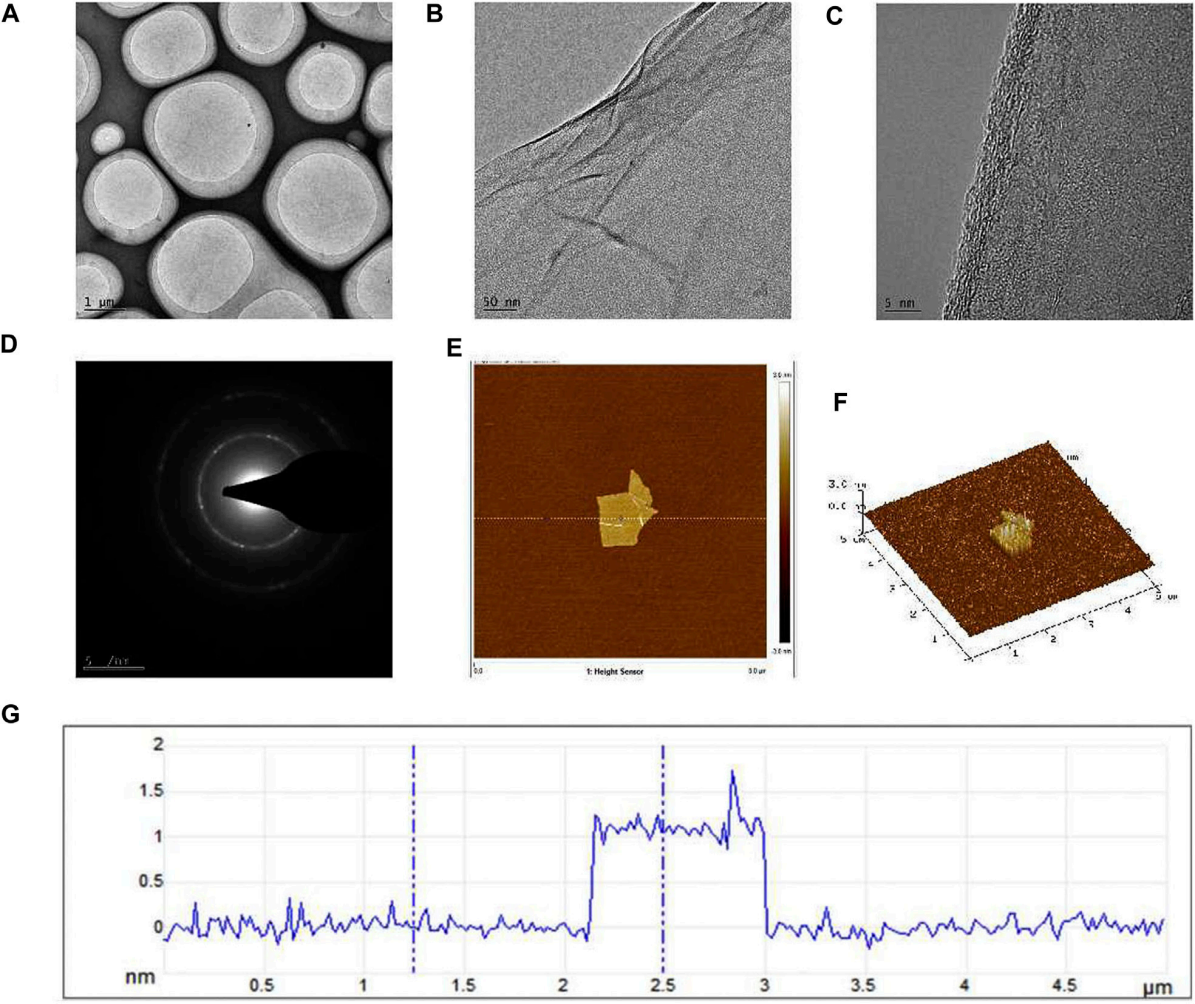
TEM and AFM images of graphene formed after oxidation-reduction reaction in this research. **(A)** The TEM image of graphene under the light field, **(B)** The folds formed in some regions of the sample were visible under the TEM, **(C)** TEM high-resolution lattice image, **(D)** The electron diffraction pattern, **(E)** AFM image of graphene, **(F)** The graphene thickness was observed by AFM, **(G)** The height curve of the underlined area.

Graphene is a single-layer carbon nanomaterial composed of sp^2^ hybrid carbon atoms with excellent conductivity and mechanical properties. In this study, we employed a method involving the oxidation and reduction of graphite to obtain single-layer sheet graphene with a regular crystal structure and observe the morphology. The TEM image of graphene under the light field is shown in Figure 1A, exhibiting a flat and continuous film suspended on a copper support grid. Furthermore, the transparent structure of graphene (Figure 1B), indicated that the thickness of our sample is very thin, and wrinkles can be observed in some regions of the sample, which is caused by the superposition of graphene sheets or the coiling of the edge zone. From the high-resolution lattice image (Figure 1C), it can be seen that the surface texture of graphene is obvious, flat and ordered, and presents a single-layer sheet structure. The electron diffraction pattern (Figure 1D) showed hexagonal diffraction spots, indicating that the lattice structure of the graphene sample was relatively complete. As depicted in the AFM image (Figure 1E), the graphene size is between 2–3 μm, with a thickness closed to 1 nm (Figures 1F, G), validating the successful isolation of a single layer of graphene, characterized by a flat and uniform surface.

### Physicochemical properties of composite tubes

**FIGURE 2:**
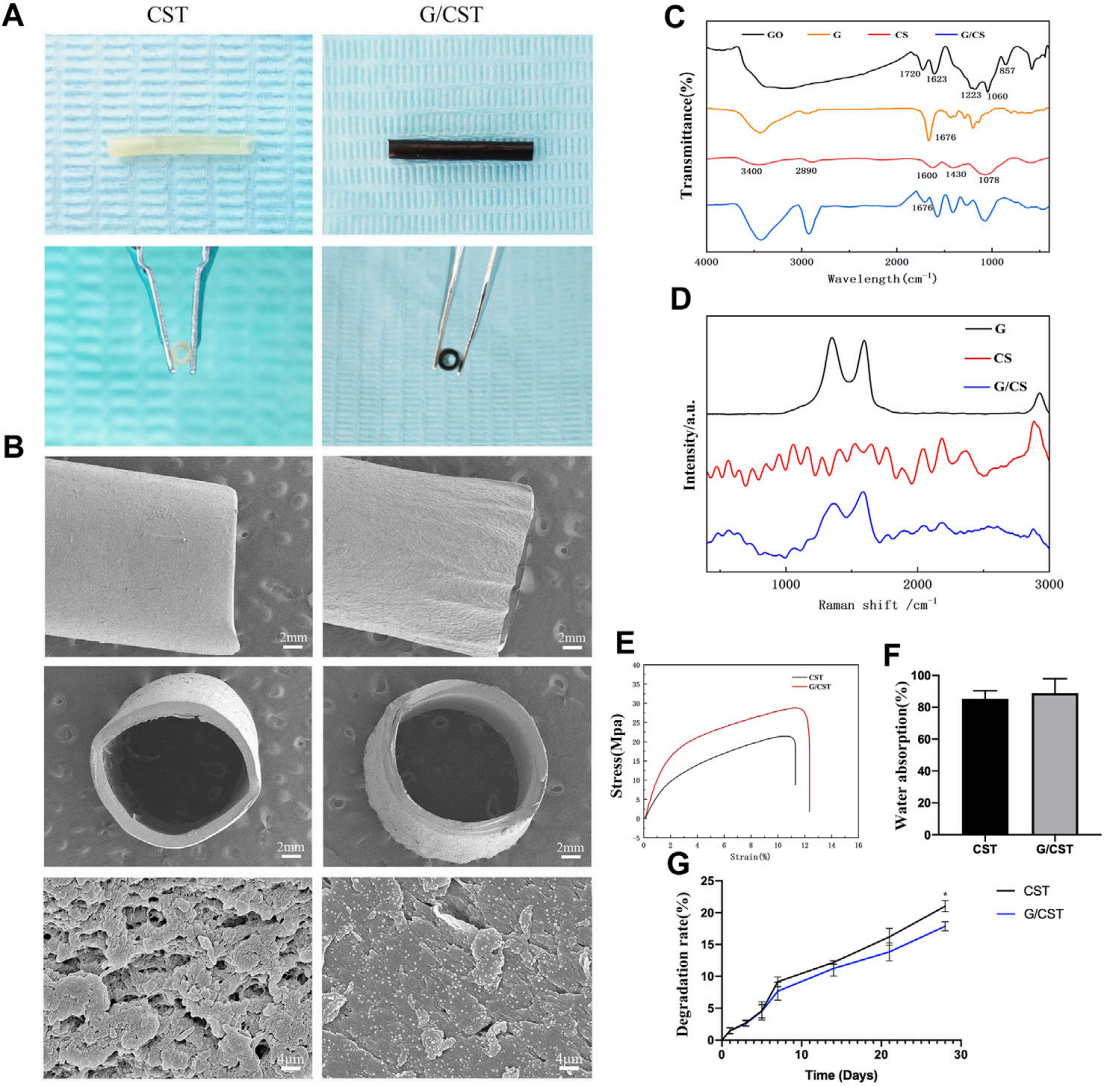
Physicochemical Properties of CST and G/CST. **(A)** The photographs of CST and G/CST, **(B)** SEM results of the CST and G/CST (up: longitudinal dimension; middle: cross section; down: high-magnification images), **(C)** FTIR spectrum of GO, G, CS and G/CS, **(D)** Raman spectra of CS and G/CS, **(E)** Stress-strain curve of CST and G/CST, **(F)** Water absorption of CST and G/CST, **(G)** The degradation rate of CST and G/CST.. (SEM and TEM represent scanning electron microscopy and transmission electron microscopy respectively; CS, G, G/CS and GO respectively stand for: chitosan, graphene, chitosan composite with graphene and graphene oxide).

On that basis, the composite tubes G/CST were prepared as described above by mixing graphene as reinforcement material with CS in this study, and their morphology, physical and chemical properties were observed. The photographs (Figure 2A) and SEM results (Figure 2B) showed that the CST and G/CST prepared exhibited favorable morphology and lumen structure, with smooth surfaces devoid of significant collapse or cracks. High-magnification images revealed uniform distribution of graphene particles on the tube surface (Figure 2B).

FTIR (Figure 2C) and Raman spectroscopy (Figure 2D) were used to analyze the composition of the tubes. The FTIR (Figure 2C) showed that, the absorption peak appeared near 1720 cm^-1^ in the middle frequency region, which was attributed to the carbanyl group (C=O) stretching vibration of carboxylic acid and carbonyl group at the edge of GO, and it belonged to the C-O-C stretching vibration of GO surface near 1223 cm^-1^, the peak near 3,430 cm^-1^ belonged to the stretching vibration of hydroxyl group (-OH), and GO had a broad spectrum peak near this region, which was derived from the water molecules adsorbed by GO. Therefore, The absorption peak of the bending vibration at 1,623 cm^−1^, which represented the water molecule -OH, was also strong. With the reduction reaction, most of the oxygen-containing absorption peaks gradually weakened or flattened, indicating that GO had been reduced. The absorption peak caused by C-OH vibration at 3,430, 1,676 and 1045 cm^−1^ was still present, the possible reason was that the reduced product still contains some oxygen groups, or water or hydroxyl groups adsorbed on the surface of the sample. Additionally, the FTIR spectra indicated the successful combination of CS and graphene. For CS, a broad band spanning from 3,000 to 3,500 cm^−1^ was observed, attributable to the overlapping vibrational spectra of the -OH and the specific spectrum of the amine group (-NH_2_) in the material. After the recombination of graphene and CS, the peak spectrum around 3,000 to 3,500 cm^−1^ became wider and stronger, indicating that after the combination of graphene and CS, some oxygen-containing group stretching vibration appeared superposition, while the peak around 1,676 cm^−1^ weakened. This indicates that some unreduced oxygen-containing groups on the surface of graphene form hydrogen bonds with CS, which marks the successful binding of graphene and CS. Raman spectra (Figure 2D) showed the characteristic peaks of graphene: The D peak (1,350 cm^−1^) represents the sp^3^ hybrid structure or edge defect and the G peak (1,580 cm^−1^) generated by the vibration of sp^2^ carbon atom. It can be seen from the figure that after reduction, the obtained graphene still exhibited certain defects. The Raman spectrum of CS has no significant wave peak, after combined with the graphene, the composite has two obvious graphene characteristic peaks around 1,350 and 1,580 cm^−1^, however, compared with graphene, the peak value of the composite was relatively weakened and widened, indicating that CS and graphene were successfully combined. The result revealed prominent characteristic peaks in our graphene oxide (GO) and graphene samples. After the reduction reaction, the degree of graphene reduction was high, with evident two-dimensional crystal characteristics, the curve after the combination of the graphene and CS can still see the characteristic peak of graphene, but the peak will be weakened or widened, which may be due to the surface of reduced graphene still has oxygen-containing groups, which can form chemical bonds with the amino group or hydroxyl group on the surface of chitosan, allowing for successful integration with CS. The effective combination of the two is conducive to the formation of a smooth and uniform nerve conduit, and enhance the mechanical properties of the conduit.

Subsequently, the mechanical properties, water absorption and degradation of the composite tubes were tested. Figure 2E showed that CST and G/CST had good mechanical properties, with the addition of graphene, the tensile strength of CST was further enhanced, increased from 21.51 to 28.86 MPa and they had a relatively stable stress level, consistent with those of the spectral analysis described above. Furthermore, water absorption (Figure 2F) test demonstrated that G/CST exhibited a favorable water absorption around 88.75% ± 7.45%, and there was no significant difference in which compared with CST group, whose water absorption rate is 85.39% ± 4.1%, it is proved that the material we prepared has good hydrophilicity, which is conducive to maintaining its own stability in liquid and is conducive to neural cell survival. Moreover, the degradation rate of G/CST was lower than that of CST (Figure 2G), CS, as an organic polysaccharide, has a large number of hydrophilic groups on its surface, so it has good hydrophilia. When the simple CST contacts with water, the polymer network is further expanded, so the degradation rate is faster. After adding graphene, graphene and CS form molecular bonds (Elhami et al., 2024), slowing down the process of molecular network expansion. The mechanical strength was enhanced and the degradation rate was slowed down without affecting hydrophilicity, potentially allowing more time for the proximal end of the defective nerve to grow into the distal end. These results consist with the study of Afsharpour et al. (Wang et al., 2016; Afsharpour et al., 2023), indicate that G/CST possesses good mechanical properties, water stability and degradation stability, which are beneficial for cell growth and tissue repair. The G/CST we prepared effectively addressed the issues of poor strength and rapid degradation associated with CS, demonstrate the potential of graphene as a mechanical reinforcement material for application in tissue engineering scaffolds.

### CST and G/CST have good cytocompatibility with DPSCs

**FIGURE 3:**
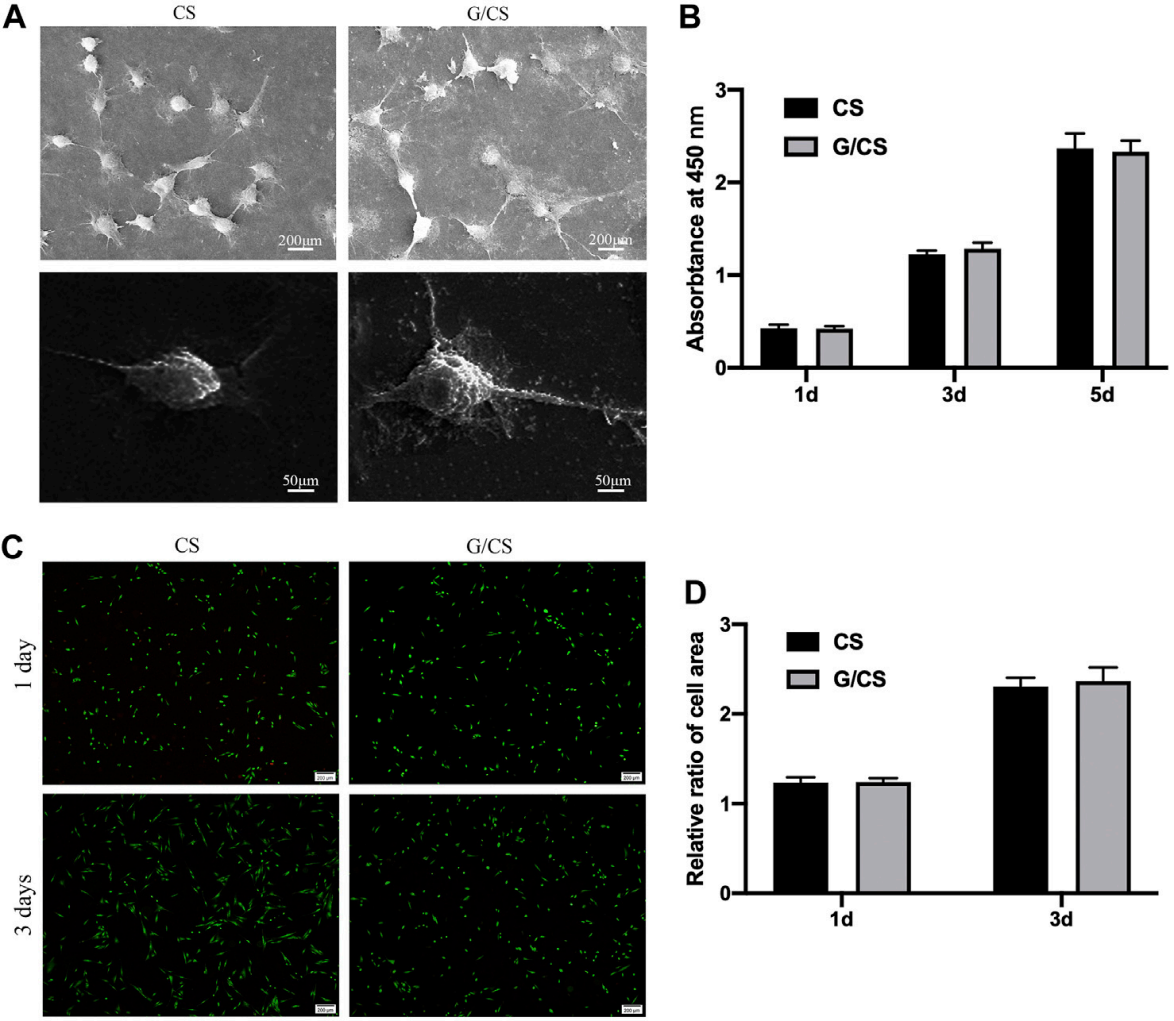
CS and G/CS membrane have good cytocompatibility with DPSCs. **(A)** SEM showed that the cells adhered well to the membrane for 24 h, with a typical long spindle cell morphology, **(B)** MTT assay showed there was no inhibitory effect on the proliferation of DPSCs on the 1st, 3rd and 5th day, with the extension of time, both groups of cells showed a proliferative state, and there was no significant difference between the two groups (*p* > 0.05). **(C)** Live/dead cell staining experiments showed that after 3 days of culture, DPSCs in both groups grew well, **(D)** Statistical analysis of living cells showed that there was no significant difference in cell number between the two groups (*p* > 0.05). The experiment was conducted utilizing analysis of variance (ANOVA), with each experiment independently replicated a minimum of three times. Results are expressed as mean ± standard deviation. Statistical significance was determined at *p* < 0.05.

The primary factor of ideal nerve conduit is good biocompatibility. The good biocompatibility of CS has been widely recognized (Li et al., 2014). Graphene as a reinforcing material is also important for the biological behavior of cells, graphene/CS composite films were cultured with DPSCs isolated from rabbit incisors (Isolation and identification methods and results are described in the Supplementary Material.) to evaluate their cytocompatibility. As shown in Figure 3, CS and G/CS had good cell compatibility, which have the potential to prepare facial nerve graft. This is consistent with the findings of Li et al. (Guo et al., 2016).

### G/CST and DPSCs promoted facial nerve regeneration and remyelination

Graphene can not only promote the proliferation of neural stem cells, but also have a certain nerve repair function (Tang et al., 2013; Li et al., 2016). Li et al. also suggested that graphene-related hydrogels could effectively promote the adhesion and proliferation of Schwann cells, and the cells showed a higher ability to release biological factors (Serrano et al., 2014; Shah et al., 2014). Incorporating a graphene nano-coating within the nerve repair conduit has been shown to enhance peripheral nerve regeneration, underscoring the significant potential of graphene-based nanotechnology in peripheral nerve repair (Qian et al., 2018; Wang et al., 2019). In this study, graphene was added to CS as a reinforcing material to prepare the composite tubes G/CST. Compared with CST, G/CST exhibits enhanced mechanical properties, good cytocompatibility, good water absorption and stable degradation, these properties make it suitable for further investigation *in vivo* for the repair of facial nerve injuries.

**FIGURE 4:**
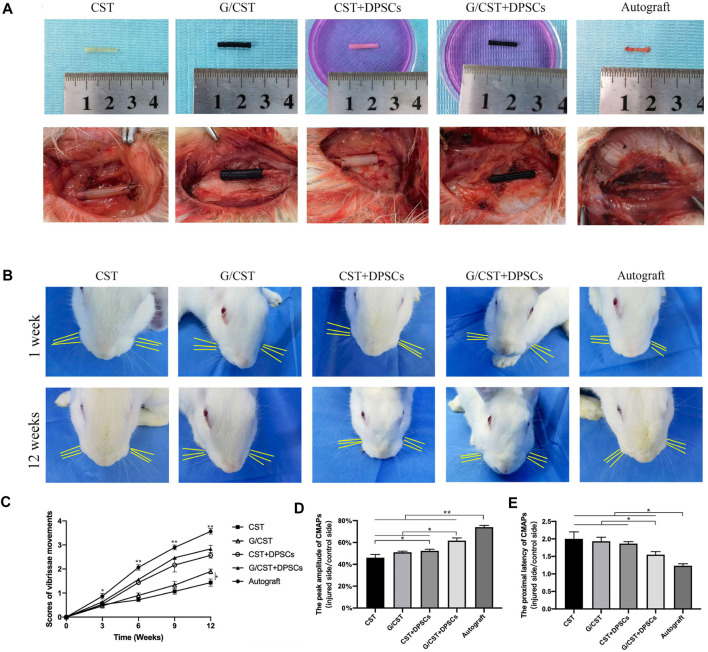
G/CST and DPSCs Promoted Recovery of Facial Nerve Function. **(A)** *In vitro* (up) and *in vivo* (down) observation of nerve grafts, **(B)** Observation of the direction of the beard at 1week and 12weeks, **(C)** Whisker movement analysis at different time points, **(D)** Peak amplitude of CMAPs, **(E)** Proximal latency of CMAPs. *p < 0.05, **p < 0.01, (n = 5).

The neural tube is lack of active functional components, and the promoting effect on nerve regeneration needs to be improved. Stem cell transplantation technology is a good solution. Schwann cells are crucial for nerve injury repair and regeneration (Gong et al., 2014; Sun et al., 2018). However, the repair efficacy of endogenous Schwann cells is limited, the application of exogenous Schwann cells or seed cells with similar functions may prove beneficial for injury repair (Heng et al., 2016; Kristjan and Rhona, 2019). Among various cell types, DPSCs have emerged as one of the most promising candidates (Martens et al., 2013a; Ullah et al., 2016) and they have been demonstrated as an ideal stem cell type for neural regeneration (Mu et al., 2022). Then, in this study, DPSCs were seeded into the G/CST and applied to rabbit facial nerve injuries (Figure 4A), the facial whisker direction (Figure 4B), the whisker movement analysis (Figure 4C), and the electrophysiological detection technology (Figures 4D, E) demonstrated that the G/CST combined with DPSCs effectively repaired facial nerve injuries, the functional recovery is better than the other three groups, but not as good as the Autograft group (For the analysis of the recovery of rabbit facial nerve function, please see Supplementary Material 2.2).

After the functional evaluation of the regenerated nerve, the regenerated nerve-like tissues were taken and fixed for histological observation. As shown in Supplementary Figure S2, HE results and IHC analysis showed that the combination of CST and DPSCs could increase the number of nerve fibers, facilitated myelin sheath regeneration, and increase the number of Schwann-like cells (Wang and Li, 2017; Kister and Kister, 2023). Hence, we proposed that G/CST + DPSCs promote the regeneration of two types of nerve cells—axon cells and Schwann-like cells, which is consistent with functional evaluations and histological observations of the regenerated nerves. DPSCs have strong neuroprotective and neurogenic functions (Guo et al., 2020; Luo et al., 2020), making them promising candidates for cell-based therapies in peripheral nerve injury treatment (Martens et al., 2013b; Martens et al., 2014). However, the mechanism of the use of composite remains to be explored. Does DPSCs differentiate into Schwann cells (Al-Zer et al., 2015; Mohan and Ramalingam, 2020), or cell differentiation play an important role that the G/CST and DPSCs create a conducive repair microenvironment (Cai et al., 2017; Kolar et al., 2017) that sustains the proliferation and differentiation capabilities of seed cells while fostering the regeneration and maturation of myelin sheaths and axons (Ullah et al., 2017; Luo et al., 2018) need to be explored. In the present study, we hypothesized that the DPSCs seeded in the G/CST could differentiate into Schwann-like cells in the excellent regeneration microenvironment created by the composite tubes promotes wound healing, suggesting that the prepared G/CST have excellent properties and is a promising material for tissue engineering nerve conduit, the combination of DPSCs and G/CST provides a therapeutic idea for the repair of facial nerve injury.

Additionally, several limitations were encountered in this study. Firstly, the conduit wall utilized in this study featured a relatively dense structure. Prior to application, we manually introduced holes in the side wall of the conduit to facilitate material exchange at the injured site. Although additional pores gradually formed with the degradation of CS, where remains room for further improvement in this regard. Moreover, while graphene boasts excellent conductivity and drug-loading properties, these advantageous features were not fully utilized in the present study. These deficiencies may have contributed to the treatment’s inability to match the efficacy of autologous nerve repair. Secondly, the method of delivering DPSCs involved implanting cells into the hollow conduit *via* artificial rotation, resulting in significant cell loss. At the same time, the retention and survival rate of cells after transplantation into vivo are low, which is also the main reason for the unsatisfactory efficacy of stem cell technology at present (Traverse et al., 2012). To address these issues, we have tried to investigate the use of a sponge as a cell carrier to fill the nerve conduit, and consider adding cytokines for immunomodulatory or neuroprotective effects (Drowley et al., 2010), with ongoing research aimed at optimizing this approach and anticipating further results. Moving forward, we aim to address these limitations through continued exploration in related fields. Further investigations into the underlying mechanisms are anticipated to provide a comprehensive understanding of the function of G/CST containing DPSCs, enriching the fundamental theory of G/CST-mediated facial nerve injury repair mechanisms. Ultimately, these insights will inform the development of novel strategies for clinical applications in facial nerve injury repair.

## Conclusion

In this study, for the first time, the mechanically reinforced composite tubes G/CST with excellent biocompatibility combined with DPSCs has a significant repair effect on facial nerve injury. This success underscores the potential of G/CST as a tissue-engineered nerve conduit and validates the effectiveness of DPSCs in facial nerve injury repair. These findings not only provide a theoretical foundation but also offer innovative repair strategies for tissue engineering approaches aimed at facial nerve injury repair. However, there are still some shortcomings in this study. In the future, we will conduct in-depth research on the microstructure of the conduit, the loading mode of cells and the mechanism of the composite conduit. At the same time, we will try to make full use of the electrical conductivity of graphene and maximize the advantages of the composite conduit of graphene and chitosan.

## Data Availability

The original contributions presented in the study are included in the article/Supplementary Material, further inquiries can be directed to the corresponding authors.

## References

[B1] Afsharpour M. Radmanesh L. Yang C. (2023). *In situ* synthesis of doped bio-graphenes as effective metal-free catalysts in removal of antibiotics: effect of natural precursor on doping, morphology, and catalytic activity. Switzerl. 28 (20), 7212. 10.3390/molecules28207212 PMC10608900 37894691

[B2] Al-Zer H. Apel C. Heiland M. Friedrich R. E. Jung O. Kroeger N. (2015). Enrichment and schwann cell differentiation of neural crest-derived dental pulp stem cells. Vivo 29 (3), 319–326. PMID: 25977377. 25977377

[B3] Cai S. Tsui Y. P. Tam K. W. Shea G. K. Chang R. S. Ao Q. (2017). Directed differentiation of human bone marrow stromal cells to fate-committed schwann cells. Stem Cell Rep. 9 (4), 1097–1108. 10.1016/j.stemcr.2017.08.004 PMC5639182 28890164

[B4] Cai Z. Chen L. Yu X. Yagoub A. E. A. Okonkwo C. E. Zhou C. (2022)). Effect of molecular weight of chitosan on the formation and properties of zein-nisin-chitosan nanocomplexes. Carbohydr. Polym. 292, 119664. 10.1016/j.carbpol.2022.119664 35725207

[B5] Cong M. Wu X. Zhu L. Gu G. Ding F. Li G. (2024). Anisotropic microtopography surface of chitosan scaffold regulating skin precursor-derived Schwann cells towards repair phenotype promotes neural regeneration. Regen. Biomater. 11 (Jan 27), rbae005. 10.1093/rb/rbae005 38414797 PMC10898340

[B6] Drowley L. Okada M. Beckman S. Vella J. Keller B. Tobita K. (2010). Cellular antioxidant levels influence muscle stem cell therapy. Mol. Ther. 18 (10), 1865–1873. 10.1038/mt.2010.160 20664528 PMC2951566

[B7] Elhami N. Pazhang M. Beygi-Khosrowshahi Y. Dehghani A. (2024). Development of nanocomposites based on chitosan/reduced graphene oxide for wound healing application. Int. J. Biol. Macromol. 258 (Pt 1), 128832. 10.1016/j.ijbiomac.2023.128832 38128799

[B8] Gong L. Zhu Y. Xu X. Li H. Guo W. Zhao Q. (2014). The effects of claudin 14 during early Wallerian degeneration after sciatic nerve injury. Neural Regen. Res. 9 (24), 2151–2158. 10.4103/1673-5374.147946 25657736 PMC4316448

[B9] Grijalvo S. Díaz D. D. (2021). Graphene-based hybrid materials as promising scaffolds for peripheral nerve regeneration. Neurochem. Int. 147, 105005. 10.1016/j.neuint.2021.105005 33667593

[B10] Guo R. Zhang S. Xiao M. Qian F. He Z. Li D. (2016). Accelerating bioelectric functional development of neural stem cells by graphene coupling: implications for neural interfacing with conductive materials. Biomaterials 106, 193–204. 10.1016/j.biomaterials.2016.08.019 27566868

[B11] Guo S. Redenski I. Landau S. Szklanny A. Merdler U. Levenberg S. (2020). Prevascularized scaffolds bearing human dental pulp stem cells for treating complete spinal cord injury. Adv. Healthc. Mater 9 (20), e2000974. 10.1002/adhm.202000974 32902147

[B12] Heng B. C. Lim L. W. Wu W. Zhang C. (2016). An overview of protocols for the neural induction of dental and oral stem cells *in vitro* . Tissue Eng. Part B Rev. 22 (3), 220–250. 10.1089/ten.TEB.2015.0488 26757369

[B13] Hou Y. Wang X. Wang Y. Chen X. Wei B. Zhang J. (2023). Electrospun nanofibrous conduit filled with a collagen-based matrix (ColM) for nerve regeneration. Molecules 28 (22), 7675. 10.3390/molecules28227675 38005397 PMC10675555

[B14] Jafarisavari Z. Ai J. Abbas Mirzaei S. Soleimannejad M. Asadpour S. (2024)). Development of new nanofibrous nerve conduits by PCL-Chitosan-Hyaluronic acid containing Piracetam-Vitamin B12 for sciatic nerve: a rat model. Int. J. Pharm. 655, 123978. 10.1016/j.ijpharm.2024.123978 38458406

[B15] Kister A. Kister I. (2023). Overview of myelin, major myelin lipids, and myelin-associated proteins. Front. Chem. 10, 1041961. 10.3389/fchem.2022.1041961 36896314 PMC9989179

[B16] Kolar M. K. Itte V. N. Kingham P. J. Novikov L. N. Wiberg M. Kelk P. (2017). The neurotrophic effects of different human dental mesenchymal stem cells. Sci. Rep. 7 (1), 12605. 10.1038/s41598-017-12969-1 28974767 PMC5626751

[B17] Kristjan R. J. Rhona M. (2019). The success and failure of the schwann cell response to nerve injury. Front. Cell. Neurosci. 13, 33. 10.3389/fncel.2019.00033 30804758 PMC6378273

[B18] Li G. Zhao Y. Zhang L. Gao M. Kong Y. Yang Y. (2016). Preparation of graphene oxide/polyacrylamide composite hydrogel and its effect on Schwann cells attachment and proliferation. Colloids Surf. B Biointerfaces 143, 547–556. 10.1016/j.colsurfb.2016.03.079 27058512

[B19] Li Y. Feng L. Shi X. Wang X. Yang Y. Yang K. (2014). Surface coating-dependent cytotoxicity and degradation of graphene derivatives: towards the design of non-toxic, degradable nano-graphene. Small 10 (8), 1544–1554. 10.1002/smll.201303234 24376215

[B20] Luo L. He Y. Jin L. Zhang Y. Guastaldi F. P. Albashari A. A. (2020)). Application of bioactive hydrogels combined with dental pulp stem cells for the repair of large gap peripheral nerve injuries. Bioact. Mater 6 (3), 638–654. 10.1016/j.bioactmat.2020.08.028 33005828 PMC7509005

[B21] Luo L. He Y. Wang X. Key B. Lee B. H. Li H. (2018). Potential roles of dental pulp stem cells in neural regeneration and repair. Stem Cells Int. 2018, 1–15. 10.1155/2018/1731289 PMC5964589 29853908

[B22] Markiewicz M. R. Callahan N. Miloro M. (2021). Management of traumatic trigeminal and facial nerve injuries. Oral Maxillofac. Surg. Clin. North Am. 33 (3), 381–405. 10.1016/j.coms.2021.04.009 34116905

[B23] Martens W. Bronckaers A. Politis C. Jacobs R. Lam- brichts I. (2013b). Dental stem cells and their promising role in neural regeneration: an update. Clin. Oral Investig. 17 (9), 1969–1983. 10.1007/s00784-013-1030-3 23846214

[B24] Martens W. Bronckaers A. Politis C. Lambrichts I. (2013a). Dental stem cells and their promising role in neural regeneration: an update. Clin. Oral Invest. 17, 1969–1983. 10.1007/s00784-013-1030-3 23846214

[B25] Martens W. Sanen K. Georgiou M. Struys T. Bronckaers A. Ameloot M. (2014). Human dental pulp stem cells can differentiate into Schwann cells and promote and guide neurite outgrowth in an aligned tissue-engineered collagen construct *in vitro* . FASEB J. 28 (4), 1634–1643. 10.1096/fj.13-243980 24352035 PMC4046066

[B26] Modrak M. Talukder M. A. H. Gurgenashvili K. Noble M. Elfar J. C. (2020). Peripheral nerve injury and myelination: potential therapeutic strategies. J. Neurosci. Res. 98 (5), 780–795. 10.1002/jnr.24538 31608497 PMC7072007

[B27] Mohan S. P. Ramalingam M. (2020). Dental pulp stem cells in neuroregeneration. J. Pharm. Bioallied Sci. 12 (Suppl. 1), S60–S66. 10.4103/jpbs.JPBS_229_20 33149432 PMC7595495

[B28] Mu X. Liu H. Yang S. Li Y. Xiang L. Hu M. (2022). Chitosan tubes inoculated with dental pulp stem cells and stem cell factor enhance facial nerve-vascularized regeneration in rabbits. ACS omega 7 (22), 18509–18520. 10.1021/acsomega.2c01176 35694480 PMC9178771

[B29] Mu X. Sun X. Yang S. Pan S. Sun J. Niu Y. (2021). Chitosan tubes prefilled with aligned fibrin nanofiber hydrogel enhance facial nerve regeneration in rabbits. ACS Omega 6 (40), 26293–26301. 10.1021/acsomega.1c03245 34660988 PMC8515574

[B30] Novoselov K. S. Geim A. K. Morozov S. V. Jiang D. Zhang Y. Dubonos S. V. (2004). Electric field effect in atomically thin carbon films. Science 306, 666–669. 10.1126/science.1102896 15499015

[B31] Pan D. Mackinnon S. E. Wood M. D. (2020). Advances in the repair of segmental nerve injuries and trends in reconstruction. Muscle Nerve 61 (6), 726–739. 10.1002/mus.26797 31883129 PMC7230025

[B32] Qian Y. Zhao X. Han Q. Chen W. Li H. Yuan W. (2018). An integrated multi-layer 3D-fabrication of PDA/RGD coated graphene loaded PCL nanoscaffold for peripheral nerve restoration. Nat. Commun. 9 (1), 323. 10.1038/s41467-017-02598-7 29358641 PMC5778129

[B33] Radwan A. M. Boxx C. Zuniga J. (2019). Post-Traumatic injuries of the trigeminal and facial nerve. Atlas oral Maxillofac. Surg. Clin. N. Am. 27 (2), 127–133. 10.1016/j.cxom.2019.05.009 31345488

[B34] Saez D. M. Sasaki R. T. Martins D. O. Chacur M. Kerkis I. da Silva M. C. P. (2019). Rat facial nerve regeneration with human immature dental pulp stem cells. Cell Transpl. 28 (12), 1573–1584. 10.1177/0963689719854446 PMC6923557 31462071

[B35] Şahin M. M. Cayonu M. Dinc S. K. Ozkocer E. Ilhan M. Uzunoğlu E. (2022). Effects of chitosan and platelet-rich plasma on facial nerve regeneration in an animal model. Eur. Arch. Otorhinolaryngol. 279 (2), 987–994. 10.1007/s00405-021-06859-6 33956207

[B36] Serrano M. C. Patiño J. García-Rama C. Ferrer M. L. Fierro J. L. G. Tamayo A. (2014). 3D free-standing porous scaffolds made of graphene oxide as substrates for neural cell growth. J. Mater Chem. B 2 (34), 5698–5706. 10.1039/c4tb00652f 32262203

[B37] Shah S. Yin P. T. Uehara T. M. Chueng S. T. Yang L. Lee K. B. (2014). Guiding stem cell differentiation into oligodendrocytes using graphene-nanofiber hybrid scaffolds. Adv. Mater 26 (22), 3673–3680. 10.1002/adma.201400523 24668911 PMC4048813

[B38] Sultan N. Amin L. E. Zaher A. R. Grawish M. E. Scheven B. A. (2020)). Neurotrophic effects of dental pulp stem cells on trigeminal neuronal cells. Sci. Rep. 10 (1), 19694. 10.1038/s41598-020-76684-0 33184395 PMC7665001

[B39] Sun Z. Wei W. Liu H. Ma J. Hu M. Huang H. (2018). Acute response of neurons: an early event of neuronal cell death after facial nerve injury. World Neurosurg. 109, e252–e257. 10.1016/j.wneu.2017.09.157 28987828

[B40] Takezawa K. Townsend G. Ghabriel M. (2018). The facial nerve: anatomy and associated disorders for oral health professionals. Odontology 106 (2), 103–116. 10.1007/s10266-017-0330-5 29243182

[B41] Tang M. L. Song Q. Li N. Jiang Z. Huang R. Cheng G. (2013). Enhancement of electrical signaling in neural networks on graphene films. Biomaterials 34, 6402–6411. 10.1016/j.biomaterials.2013.05.024 23755830

[B42] Traverse J. H. Henry T. D. Pepine C. J. Willerson J. T. Zhao D. X. Ellis S. G. (2012). Effect of the use and timing of bone marrow mononuclear cell delivery on left ventricular function after acute myocardial infarction: the TIME randomized trial. JAMA 308 (22), 2380–2389. 10.1001/jama.2012.28726 23129008 PMC3652242

[B43] Ullah I. Park J. M. Kang Y. H. Byun J. H. Kim D. G. Kim J. H. (2017). Transplantation of human dental pulp-derived stem cells or differentiated neuronal cells from human dental pulp-derived stem cells identically enhances regeneration of the injured peripheral nerve. Stem Cells. Dev. 26 (17), 1247–1257. 10.1089/scd.2017.0068 28657463

[B44] Ullah I. Subbarao R. B. Kim E. J. Bharti D. Jang S. J. Park J. S. (2016). *In vitro* comparative analysis of human dental stem cells from a single donor and its neuronal differentiation potential evaluated by electrophysiology. Life Sci. 154 (16), 39–51. 10.1016/j.lfs.2016.04.026 27107840

[B45] Wang J. Cheng Y. Chen L. Zhu T. Ye K. Jia C. (2019). *In vitro* and *in vivo* studies of electroactive reduced graphene oxide-modified nanofiber scaffolds for peripheral nerve regeneration. Acta Biomater. 84, 98–113. 10.1016/j.actbio.2018.11.032 30471474

[B46] Wang L. Li P. (2017). Expressions of nestin and glial fibrillary acidic protein in rat retina after optic nerve transection. Int. J. Ophthalmol. 10 (10), 1510–1515. 10.18240/ijo.2017.10.05 29062768 PMC5638970

[B47] Wang T. V. Delaney S. Pepper J. P. (2016). Current state of stem cell-mediated therapies for facial nerve injury. Curr. Opin. Otolaryngology Head Neck Surg. 24 (4), 285–293. 10.1097/MOO.0000000000000292 27379549

[B48] Wu H. Zhang J. Luo Y. Wan Y. Sun S. (2015). Mechanical properties and permeability of porous chitosan-poly(p-dioxanone)/silk fibroin conduits used for peripheral nerve repair. J. Mech. Behav. Biomed. Mater 50, 192–205. 10.1016/j.jmbbm.2015.06.016 26143352

[B49] Xue C. Zhu H. Wang H. Wang Y. Xu X. Zhou S. (2023). Skin derived precursors induced Schwann cells mediated tissue engineering-aided neuroregeneration across sciatic nerve defect. Bioact. Mater. 33, 572–590. 10.1016/j.bioactmat.2023.11.016 38111651 PMC10726219

[B50] Zhang M. An H. Wan T. Jiang H. R. Yang M. Wen Y. Q. (2023). Micron track chitosan conduit fabricated by 3D-printed model topography provides bionic microenvironment for peripheral nerve regeneration. Int. J. Bioprint 9 (5), 770. 10.18063/ijb.770 37608847 PMC10339431

[B51] Zhao Y. Liu Y. Kang S. Sun D. Liu Y. Wang X. (2024a). Peripheral nerve injury repair by electrical stimulation combined with graphene-based scaffolds. Front. Bioeng. Biotechnol. 12 (Feb 28), 1345163. 10.3389/fbioe.2024.1345163 38481574 PMC10933080

[B52] Zhao Y. Liu Y. Lu C. Sun D. Kang S. Wang X. (2024b). Reduced graphene oxide fibers combined with electrical stimulation promote peripheral nerve regeneration. Int. J. Nanomedicine 19, 2341–2357. 10.2147/IJN.S449160 38469057 PMC10926921

[B53] Zhao Y. N. Wu P. Zhao Z. Y. Chen F. X. Xiao A. Yue Z. Y. (2023). Electrodeposition of chitosan/graphene oxide conduit to enhance peripheral nerve regeneration. Neural Regen. Res. 18 (1), 207–212. 10.4103/1673-5374.344836 35799544 PMC9241416

[B54] Zheng K. Feng G. Zhang J. Xing J. Huang D. Lian M. (2021). Basic fibroblast growth factor promotes human dental pulp stem cells cultured in 3D porous chitosan scaffolds to neural differentiation. Int. J. Neurosci. 131 (7), 625–633. 10.1080/00207454.2020.1744592 32186218

