# Supplementary material for: Graphene/ chitosan tubes inoculated with dental pulp stem cells promotes repair of facial nerve injury
Source: Front Chem. 2024 Jun 3;12:1417763. doi: 10.3389/fchem.2024.1417763 (PMC11180760; doi:10.3389/fchem.2024.1417763)
Supplement: Supplementary file 1 [file DataSheet1.pdf]

frontiers

Supplementary Material

1 Materials and Methods

1.1 Preparation of the Graphene Oxide (GO)

By Hummers method, graphene oxide (GO) was obtained by chemical oxidation and external force stripping of graphite (Macklin). The obtained GO was prepared into 0.5mg/mL dispersion, and hydrazine hydrate ($N_2H_4 \cdot H_2O$) was added at a ratio of 2:1 for reduction. After reduction, the graphene powder was obtained by repeated washing with deionized water and freeze-drying.

1.2 Measurement of the water absorption of CST and G/CST

The freeze-dried samples were immersed in distilled water for 24 hours, then the samples were taken out and weighed immediately after removing the water with filter paper, and the water absorption rate was measured according to the following formula: Ra= （W_2-W_1） /W_1×100%, where W_1 is the weight of the dried tubes, and W_2 is the weight of the conduits uptaking water. Five parallel samples were used for each test and three individual tests were performed.

1.3 Measurement of the degradation rate of CST and G/CST

The CSTs and G/CSTs were dried to a constant weight, weighed and recorded as W_3. The samples were immersed in 50 ml of PBS solution containing lysosome (1×10^7 U/L) and left in a water bath at 37℃. The samples were taken out at 1, 3, 5, 7, 14, 28 days, washed with deionized water repeatly, and then drying to constant weight, weighing and record the corresponding sample weight as W4, then calculate the degradation rate of tubes, computation formula is as follows: Rd = (W_3 - W_4)/W_3 × 100%. The experiment was repeated 3 times and the mean value was recorded for inclusion in the statistics.

1.4 Rabbit DPSCs Isolation and Identification

Rabbit DPSCs (rDPSCs) were isolated and cultured by tissue block method, which can be summarized as follows: the incisors and bilateral molars of New Zealand white rabbits were extracted. After cleaning with PBS solution containing double antibiotics, the pulp tissue was removed, the apical part of the pulp was removed with sterile scissors, and the pulp tissue was cut into pieces, they were cultured in DMEM complete medium containing 20% fetal bovine serum (FBS), 1% penicilin-streptomycin (10,000 U/mL) (Gibco) in an incubator at 37°C, 5% CO_2. When the cells climbed out of the tissue edge and converged to 80%, they were digested with trypsin and subsequently subcultured in complete medium containing 10% FBS.

After the passage 3-5 of rDPSCs had grown to 80%, they were digested with trypsin and seeded into 24-well plates. Then immunohistochemistry (IHC) and immunofluorescence (IF) staining were performed to identify the expression of vimentin and STRO-1. Then, the rDPSCs were induced with mineralization induction medium for 21 days. Alizarin red staining and alkaline phosphatase staining (ALP staining) were used to identify their osteogenic differentiation ability. Then the cells were induced with nerve induction solution, IF staining was performed to evaluate the rDPSCs' ability to differentiate into neural cells.

1.5 Assessment of Biocompatibility of the Synthetic Tubes *In Vitro*

SEM was used to observe the cell adhesion on the CS and G/CS film, the rDPSCs were cultured at the density of 2 x 10^4 cells on the films in DMEM with 1% of penicillin-streptomycin and 10% FBS in a humidified incubator with 5% CO_2 at 37°C for 24 h, then the samples were fixed in 2.5% glutaraldehyde for 30 min, and dehydrated through an ethanol gradient. The samples were sputter-coated with a layer of Pt for SEM imaging after freeze dring.

The 3-(4,5-dimethylthiazol-2-yl)-2,5 diphenyl tetrazolium bromide (MTT) assay was used to evaluate the proliferation of cells cultured on the CS and G/CS films to detect the cytocompatibility of G/CS film. Briefly, the DPSCs were cultured at the density of 8 x 10^3 cells on the film in DMEM with 1% of penicillin-streptomycin and 10% FBS in a humidified incubator with 5% CO_2 at 37°C. At each time point (1, 3, and 5 days after cells seeding), the absorption was measured according to the instruction and statistically analyzed.

Calcein-AM/PI Double Stain Kit (Yeasen) was used for cell viability assay. The cells were seeded on CS and G/CS membranes at a cell density of 5 × 10^4/mL. Samples were collected on days 1 and 3, and stained according to the manufacturer's instructions. Images were acquired using a fluorescence microscope (Olympus, Japan) and quantified for viable cell area using ImageJ.

1.6 Functional Evaluation and Histological Evaluation of the Regenerated Facial Nerve

The facial muscle movement and the static and moving condition of the beard on the surgical side were observed and recorded by two non-participants who were blinded to the study every 3 weeks, the following criteria were used for assessment: 0, no significant movement; 1, barely detectable movement; 2, less significant spontaneous movement; 3, significant but asymmetric spontaneous movement, and 4 for spontaneous movement symmetrical to the healthy side. The muscle complex action potentials (CMAPs) were recorded to evaluate the electrophysiological function of regenerated nerves at 12 weeks after surgery, the peak amplitude and latency of CMAPs were then calculated for statistical analysis (n = 5). Subsequently, the facial nerve at the surgical site was collected and fixed for histological observation. 12 weeks after surgery, the regenerated nerve tissues from the surgical area were observed by hematoxylin eosin staining (HE) and TEM. The specific proteins regenerated nerve were identified by IHC. The nerve tissue were fixed with 4% paraformaldehyde. After longitudinal and transverse incision, HE staining, anti-Nestin and S-100 IHC staining were performed to observe the density of regenerated nerve tissue and the expression of specific proteins according to the procedure. Another part of the middle segment of the regenerated

facial nerve were fixed with 2% glutaraldehyde, then the tissues were subsequently fixed with 1% osmic acid, after gradient dehydration with ethanol, they were embedded in epoxy resin, and the regenerated axon density and myelin sheath thickness were observed by ultrathin sections (70nm) by TEM. All images are analyzed using Image J.

2 Results and Discussion

2.1 Isolation and culture of dental pulp stem cells (DPSCs)

In this study, The rabbit incisors were completely extracted as shown in Figure S1A. The cells were isolated from dental pulp tissue of the rabbits teeth for primary culture, as shown in Figure S1B, from the 5-10 days, cells were observed around the tissue with short spindle shape, morphology typical of mesenchymal stem. Moreover, Vimentin (Figure S1C) and STRO-1(Figure S1F) were positively expressed in the cells isolated from rabbit dental pulp in this study. After mineralization for 14 days, alizarin red (Figure S1D) and ALP (Figure S1E) were positively expressed, and after induction of neural differentiation, neural markers Nestin and NF200 were both positively expressed (Figure S1G), indicating that the cells isolated in this study had the characteristics of mesenchymal stem cells and the ability of multi-directional differentiation. The above results indicate that we successfully isolated and characterized rabbit DPSCs, who possess high proliferation and multilineage differentiation potential. The DPSCs’ expression of neurospecific markers was significantly enhanced after induction with the nerve induction medium, which demonstrated their neurogenic nature and potential utility in repairing facial nerve injuries.

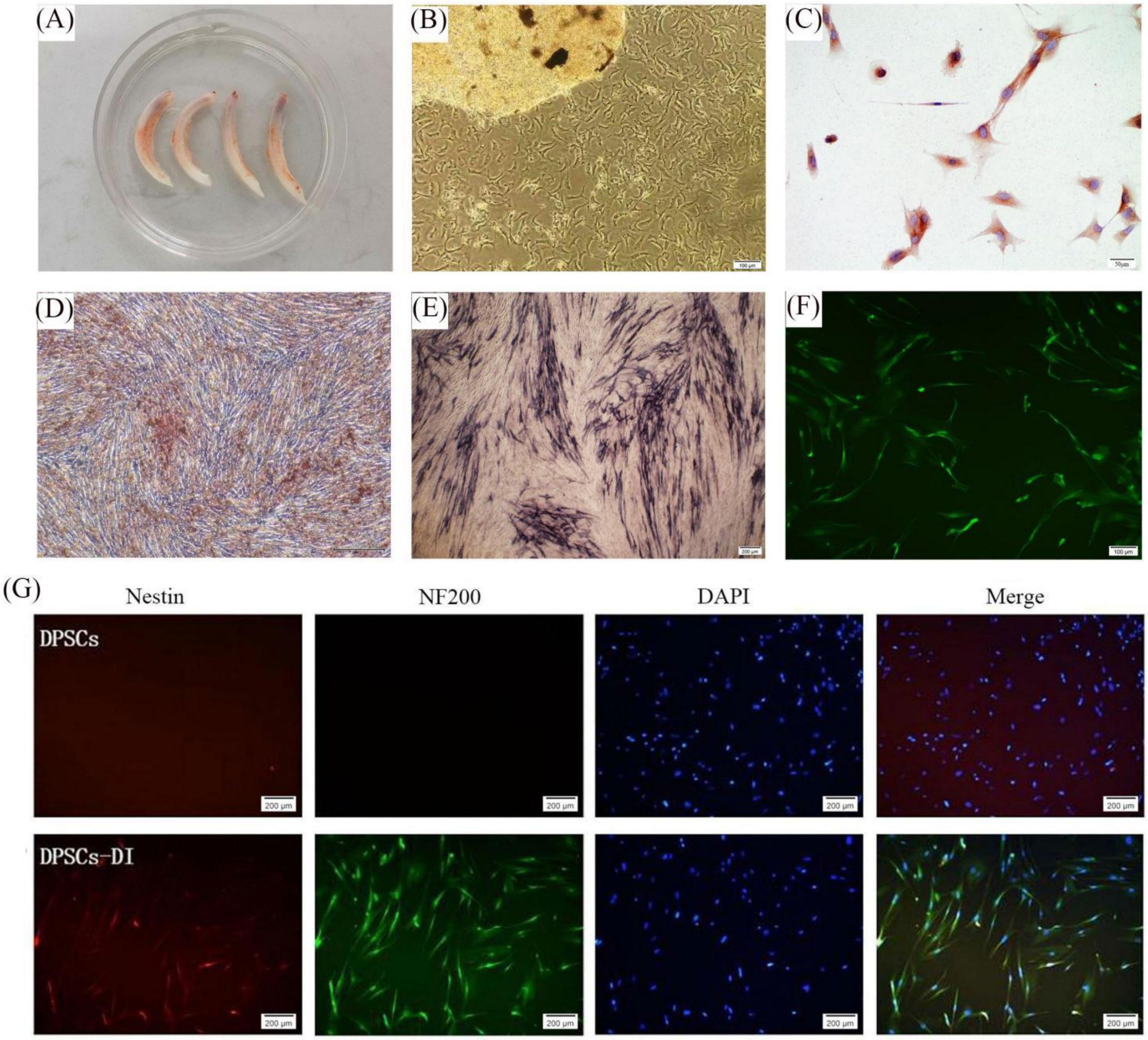

Figure S1.

Culture and Identification of DPSCs. (A) Extracted teeth of rabbits, (B) Primary culture, (C) Positive expression of vimentin, (D) Alizarin staining after 14 days of osteogenic induction, (E) Positive cells stained with ALP, (F) Positive cells of IF staining of Stro-1, (G) DPSCs (up) and DPSCs differentiated by induced fluid (down) expressed Nestin (red) and NF200 (green).

2.2 G/CST and DPSCs Promoted Recovery of Facial Nerve Function.

DPSCs were seeded into the G/CST and applied to rabbit facial nerve injuries as shown in Figure 4A. 1 week after operation, compared with the healthy side, the direction of the beard on the operated side in 5 groups was tilted to the right posterior. At 12 weeks after surgery, the facial whisker direction of the operated side was similar to that of the healthy side in Autograft group, the

CST+DPSCs group and G/CST+DPSCs group also recovered well, but there was still a certain gap compared with the healthy side, while the CST and G/CST groups recovered somewhat, but the recovery was not good (Figure 4B). Whisker movement analysis was conducted every 3 weeks (Figure 4C), the score of each group decreased to 0 after operation. 3 weeks after surgery, the scores increased in all groups, with the Autograft group exhibiting significantly higher scores compared to the other groups ($P < 0.05$). However, scores in the other groups showed no statistical difference ($P > 0.05$). At 6 weeks post-surgery, scores in the CST+DPSCs group and G/CST+DPSCs group were comparable ($P > 0.05$), yet both were significantly higher than those in the CST and G/CST groups, and lower than the Autograft group ($P < 0.01$). At 9 weeks, the whisker pulling reflex score in the G/CST+DPSCs group was notably higher than that in the CST and G/CST groups ($P < 0.01$), slightly higher than that in the CST+DPSCs group without statistical significance ($P > 0.05$), and significantly lower than the Autograft group ($P < 0.01$). The G/CST group showed slightly higher scores than the CST group, but with no statistical difference ($P > 0.05$). By 12 weeks post-surgery, the trend of scores in each group remained similar to that at 9 weeks. Notably, scores in the G/CST group were significantly higher than those in the CST group ($P < 0.05$). The difference in scores between the CST+DPSCs group and G/CST+DPSCs group remained non-significant ($P > 0.05$), but both were lower than the Autograft group ($P < 0.01$). To evaluate nerve conduction recovery, electrophysiological detection technology was used to analyze the maximum amplitude and delay time of neural action potential. CMAP waveform analysis revealed that the Autograft group exhibited the highest amplitude (Figure 4D), significantly surpassing all other groups ($P < 0.01$). Although the amplitude of the G/CST+DPSCs group differed from that of the Autograft group, it remained significantly higher than the other three groups ($P < 0.01$). The CST+DPSCs group showed a significantly higher amplitude compared to the CST group ($P < 0.05$). Regarding proximal latency (Figure 4E), the Autograft group demonstrated the shortest latency. The G/CST+DPSCs group exhibited significantly shorter latency compared to the other three groups ($P < 0.05$). Moreover, the CST+DPSCs group displayed shorter latency compared to the G/CST group and CST group ($P < 0.05$). The findings demonstrated that the G/CST combined with DPSCs effectively repaired facial nerve injuries, the facial muscle movement and electrophysiological response were recovered. The repair effect is better than the other 3 groups, but not as good as the Autograft group.

2.3 G/CST and DPSCs Promoted Facial Nerve Regeneration and Remyelination.

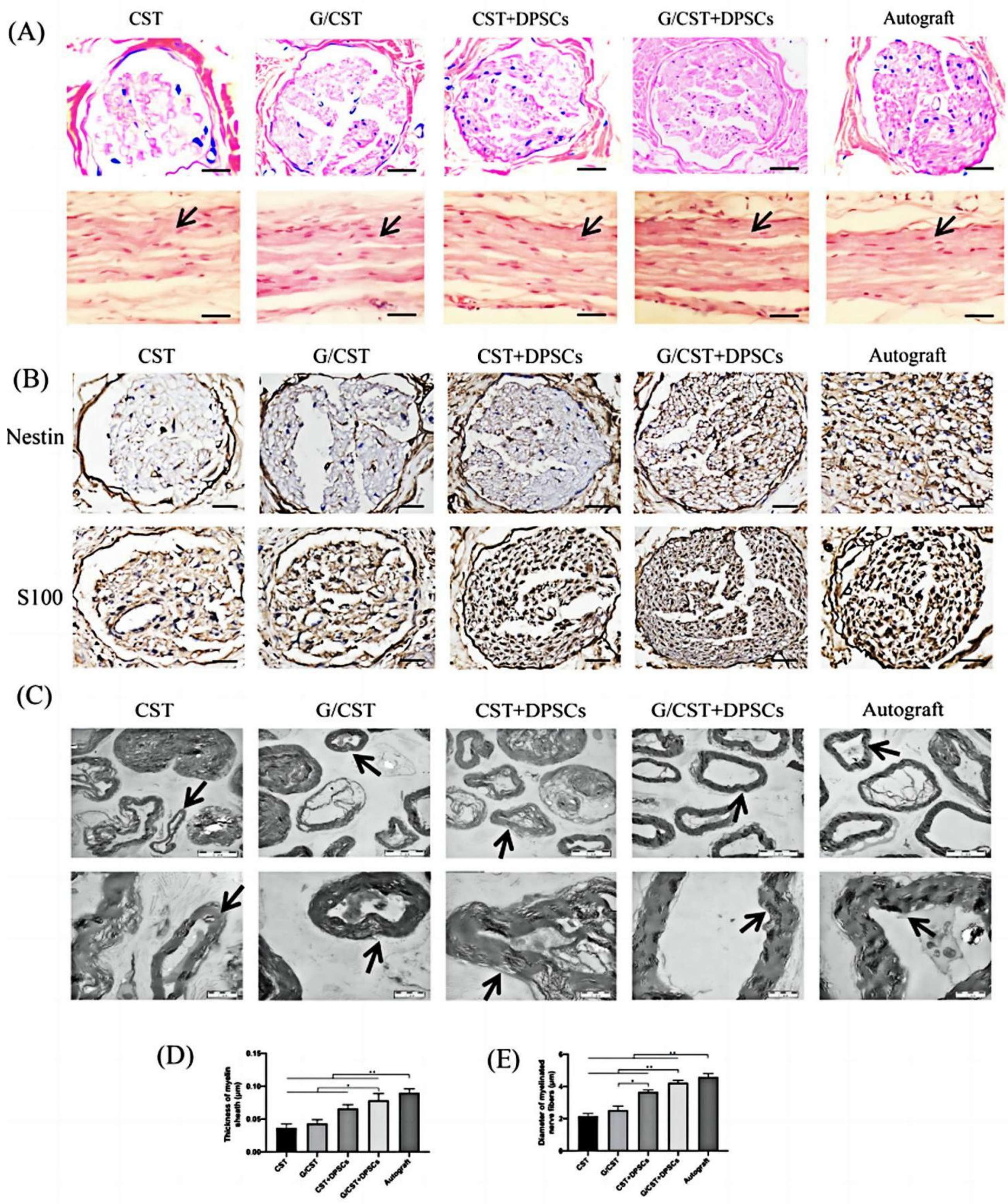
(A)
CST
G/CST
CST+DPSCs
G/CST+DPSCs
Autograft
(B)
CST
G/CST
CST+DPSCs
G/CST+DPSCs
Autograft
Nestin
S100
(C)
CST
G/CST
CST+DPSCs
G/CST+DPSCs
Autograft
(D)
Thickness of myelin sheath (μm)
0.15
0.10
0.05
0.00
CST
G/CST
CST+DPSCs
G/CST+DPSCs
Autograft
(E)
Diameter of myelinated nerve fibers (μm)
6
4
2
0
CST
G/CST
CST+DPSCs
G/CST+DPSCs
Autograft

Figure S2.

G/CST and DPSCs Promoted Facial Nerve Regeneration and Remyelination. (A) HE staining of regenerated facial nerve, transverse sections (up) showed that the Autograft group exhibited the highest density of regenerated facial nerve tissue, followed by the G/CST+DPSCs group, while the CST group showed the lowest density. The density of regenerated facial nerve tissue was comparable between the CST+DPSCs group and the G/CST group, and longitudinal sections (down) revealed that the density of regenerated facial nerve tissue in the G/CST+DPSCs group closely resembled that in the Autograft group, with the CST group exhibiting the lowest density as well. Notably, the other 3 groups displayed similar densities, Bar: 50μm, (B) IHC staining of Nestin and S100 (Bar: 50μm), (C) Observation of regenerated facial nerve by TEM: The Autograft group exhibited significantly greater diameter and thickness of the myelin sheath compared to the other groups; the myelinated nerve fibers in the CST group and G/CST group appeared loose and disordered, exhibiting irregular morphology with varying fiber diameters and thicknesses. While the arrangement of myelin sheaths in the CST+DPSCs group and G/CST+DPSCs group was relatively uniform and displayed more regular morphology compared to the CST group and G/CST group, there remained a discernible difference compared to the Autograft group, however. (D) Statistical analysis of myelin thickness, (E) Statistical analysis of myelin diameter. * $P < 0.05$, ** $P < 0.01$. The black arrow indicates the regenerated nerve tissue fibers and myelin sheath.
